# Effects of Nb Content and Heat Treatment on the Microstructure and Properties of Plasma-Sprayed CoCrFeNiNb*_x_* High-Entropy Alloy Coatings on Ductile Iron Substrates

**DOI:** 10.3390/ma19081500

**Published:** 2026-04-09

**Authors:** Kaibo Zhu, Jie Wang, Biju Zheng

**Affiliations:** Faculty of Materials Science and Engineering, Kunming University of Science and Technology, Kunming 650093, China; zhukb01@163.com (K.Z.); wangjiekust@163.com (J.W.)

**Keywords:** high-entropy alloy, atmospheric plasma spraying, ductile iron, Nb content, Laves phase, annealing treatment

## Abstract

**Highlights:**

**What are the main findings?**

- Coating hardness rises monotonically with Nb content, reaching 470.36 HV at *x* = 1.00.
- Optimal as-sprayed tribological performance occurs at Nb *x* = 0.75 with the lowest wear rate.
- 500 °C annealing achieves peak hardness (477.45 HV) and 45% lower wear rate than as-sprayed.

**What are the implications of the main findings?**

- This work provides a substrate-compatible protective coating strategy for ductile iron components.
- Nb content-annealing synergistic regulation guides wear-resistant HEA coating design.
- Low-temperature annealing avoids substrate degradation while enhancing coating performance.

**Abstract:**

Ductile iron suffers from insufficient wear resistance under heavy-load service conditions. Surface engineering technologies offer effective solutions to this problem. However, current research on the application of atmospheric plasma-sprayed (APS) CoCrFeNiNb*_x_* high-entropy alloy (HEA) coatings on ductile iron and the systematic study of compatible heat treatment processes with the substrate are still insufficient. In this study, CoCrFeNiNb*_x_* HEA coatings (*x* = 0.25, 0.50, 0.75, 1.00) were deposited on QT800-5 ductile iron by APS, and the effects of Nb content and low-temperature annealing (400–600 °C) on coating microstructure and properties were investigated. The *x* = 0.25 coating exhibited a single face-centered cubic (FCC) solid solution structure, while coatings with *x* ≥ 0.50 comprised an FCC solid solution and Cr_2_Nb-type Laves phase; hardness increased with Nb content, and as-sprayed wear resistance peaked at *x* = 0.75. Post-deposition annealing at 500 °C yielded a peak hardness of 477.45 HV and reduced the wear rate by 45% relative to the as-sprayed condition, with no measurable degradation of the substrate. These findings offer a practical reference for developing wear-resistant coatings on ductile iron components.

## 1. Introduction

Ductile iron (DI) is widely used in mining equipment, automotive drivetrains, industrial machinery, and rail systems, owing to its combination of high strength, good toughness, vibration-damping capacity, near-net-shape castability, and relatively low production cost [1,2]. In service, such components routinely experience friction, abrasive erosion, and cyclic mechanical loading, which promote surface damage in the form of abrasive wear, delamination spalling, and fatigue cracking—all of which progressively erode component reliability. Where contact stresses are high or abrasive media are present, the intrinsic wear resistance of conventional ductile iron is generally insufficient for long-term use [3,4]. To address these limitations, bulk modification methods, including alloying with Mo, Ni, Cr, and Re; reinforcement with nanoscale ceramic particles; and thermal processing routes such as quenching and tempering, have been widely studied to improve the wear resistance of DI materials [5,6]. However, these methods often face challenges due to high production costs, significant energy use, and difficulties in precise process control, which limit their wider use. Surface engineering strategies, by contrast, provide a more practical and cost-effective alternative by selectively enhancing wear resistance at the component surface without affecting the bulk material. Among these, the application of protective coatings has proven to be a particularly versatile and straightforward approach, and a range of deposition methods have been employed for this purpose [7,8,9,10,11].

Among the various coating deposition techniques available, atmospheric plasma spraying (APS) has attracted particular attention as a readily scalable, industrially viable process. It operates at high plasma temperatures, accommodates a wide range of coating materials, and readily allows adjustment of coating thickness. Its heat input to the substrate is also comparatively low; particles impact and solidify rapidly, confining thermal exposure to a thin surface layer and producing a heat-affected zone considerably narrower than those typical of laser cladding or surfacing welding. This thermal advantage is especially important for ductile iron, where excessive heat input can cause graphite nodule dissolution, matrix softening, and thermal cracking. The ability of APS to work with heat-sensitive substrates makes plasma-sprayed high-entropy alloy (HEA) coatings on ductile iron a technically and economically advantageous option for surface protection [12,13].

Building on the suitability of APS for thermally sensitive substrates, the choice of coating composition becomes the next critical consideration. Among candidate high-entropy alloy systems, the equimolar CoCrFeNi quaternary alloy is widely recognized for its stable single-phase FCC solid solution structure [14,15]. However, the inherently low yield strength of this single-phase FCC structure limits performance under high contact loads, as insufficient hardness accelerates plastic deformation and wear. Studies have shown that incorporating elements with markedly different atomic radii—such as Nb—is an effective strategy for achieving a synergistic balance of strength and ductility in CoCrFeNi-based HEAs [16,17]. The strongly negative mixing enthalpy between Nb and the constituent transition metals promotes elemental segregation during solidification, inducing significant lattice distortion (solid solution strengthening) and the formation of hard Cr_2_Nb-type Laves phases (second-phase strengthening). An optimal Nb addition therefore improves coating hardness and wear resistance while preserving adequate toughness [18,19]. The general trends of FCC → FCC + Laves phase transition with increasing Nb content, monotonic hardness increase, and peak wear resistance at intermediate Nb levels have been documented in laser-clad and cast CoCrFeNiNb*_x_* systems [17,18,19,20,21,22]. However, APS processing imposes fundamentally different thermal conditions (~10^6^ K·s^−1^) compared to laser cladding (~10^3^–10^5^ K·s^−1^), causing higher Nb supersaturation in the FCC matrix and distinct precipitation behavior upon post-spray annealing. Despite the promise of this composition–process combination, most current research on CoCrFeNiNb*_x_* coatings has focused on laser cladding onto steel or titanium substrates, primarily examining as-cast or as-clad microstructures [20,21,22]. Systematic investigation of APS-deposited CoCrFeNiNb*_x_* coatings on ductile iron remains scarce. The ultra-high cooling rate inherent to APS suppresses solute partitioning and retains a substantial excess of solute atoms in solid solution—a metastable condition unattainable by laser cladding. Consequently, Laves phase precipitation in APS coatings follows a distinctly different path than in cast or clad counterparts, and the mechanisms governing this behavior are not yet well understood.

The distinct precipitation behavior of APS coatings naturally motivates the use of post-spray annealing to drive controlled microstructural evolution. Post-spray annealing is commonly employed to relieve residual tensile stresses, close interlamellar pores, and promote precipitation reactions. However, its application to ductile iron substrates introduces a critical constraint: the annealing temperature must remain below the austenitization onset (Ac_1_). Once this threshold is crossed, the ferritic/pearlitic matrix undergoes irreversible phase transformation, permanently compromising the substrate’s mechanical properties. In practice, many reported post-treatment procedures exceed the Ac_1_ of ductile iron, and systematic low-temperature protocols specifically designed for this substrate remain scarce. This constitutes a major unresolved challenge for optimizing APS coatings on ductile iron and forms a central motivation for the present study.

To address the knowledge gaps identified above, CoCrFeNiNb*_x_* HEA coatings were deposited on QT800-5 ductile iron substrates by APS in this study. The composition that produced the best as-sprayed performance was then subjected to low-temperature stress relief annealing at 400, 500, and 600 °C—all safely below the substrate’s Ac_1_—to examine the microstructural changes and property improvements caused by heat treatment. To the best of our knowledge, the main scientific contribution of this work is the development of a substrate-compatible, low-temperature post-spray annealing process that enhances Laves-phase-mediated wear resistance in APS HEA coatings without damaging the thermally sensitive ductile iron substrate. Specifically, this study explores the following research questions: (1) How does the ultra-high cooling rate of APS affect the FCC/Laves phase balance and wear behavior as Nb content increases (*x* = 0.25–1.00); (2) What mechanisms work together to produce peak wear resistance at an intermediate Nb level; (3) How does low-temperature annealing (400–600 °C, below Ac_1_) influence the microstructure–property relationship, aiming to optimally balance Laves phase precipitation strengthening with substrate integrity.

## 2. Materials and Methods

### 2.1. Experimental Materials

QT800-5 ductile iron plates (10 mm × 10 mm × 5 mm) served as substrates. Before spraying, the substrates were sequentially ground with 200–800 grit SiC abrasive paper (Shanghai Naite Abrasive Co., Ltd., Shanghai, China), ultrasonically cleaned in anhydrous ethanol (Sinopharm Chemical Reagent Co., Ltd., Shanghai, China) for 15 min, and thoroughly dried. The feedstock powder consisted of gas-atomized spherical CoCrFeNi HEA powder (particle size: 15–45 μm; single-phase FCC, Beijing Xingrongyuan Technology Co., Ltd., Beijing, China) as the base, blended with elemental Nb powder (purity > 99.9%, Shanghai Aladdin Biochemical Technology Co., Ltd., Shanghai, China) to prepare four feedstock compositions: CoCrFeNiNb*_x_* (*x* = 0.25, 0.50, 0.75, 1.00). The subscript *x* in CoCrFeNiNb*_x_* represents the nominal atomic ratio of Nb relative to the equimolar CoCrFeNi base (i.e., designed feedstock molar ratios). The actual coating compositions may deviate from the nominal values due to in-flight oxidation and differential melting during APS. The powders were dry-mixed in a planetary ball mill (XQM-4L, Changsha Tianchuang Powder Technology Co., Ltd., Changsha, China) at room temperature with a ball-to-powder mass ratio of 3:1 and a rotational speed of 200 r·min^−1^ for 2 h. The mixture was sieved through a 325-mesh screen to remove coarse particles and vacuum-dried at 60 °C for 4 h to ensure adequate powder flowability.

### 2.2. Plasma Spraying and Heat Treatment

Prior to spraying, substrate surfaces were grit-blasted with 20–25 mesh white corundum (Zhengzhou Yufa Abrasive Group Co., Ltd., Zhengzhou, China) at a blasting pressure of 0.6–0.8 MPa and a standoff distance of 10 cm, to produce adequate roughness and remove surface contaminants. Coatings were subsequently deposited with a Metco F4MB-XL APS system (Oerlikon Metco AG, Pfäffikon, Switzerland) under the following conditions: argon flow rate 60 L·min^−1^, hydrogen flow rate 8 L·min^−1^, current 550 A, voltage 60 V, spraying distance 110 mm, and gun traverse speed 700 mm·s^−1^. Coating thickness after deposition ranged from 370 to 420 μm.

**Figure 1:**
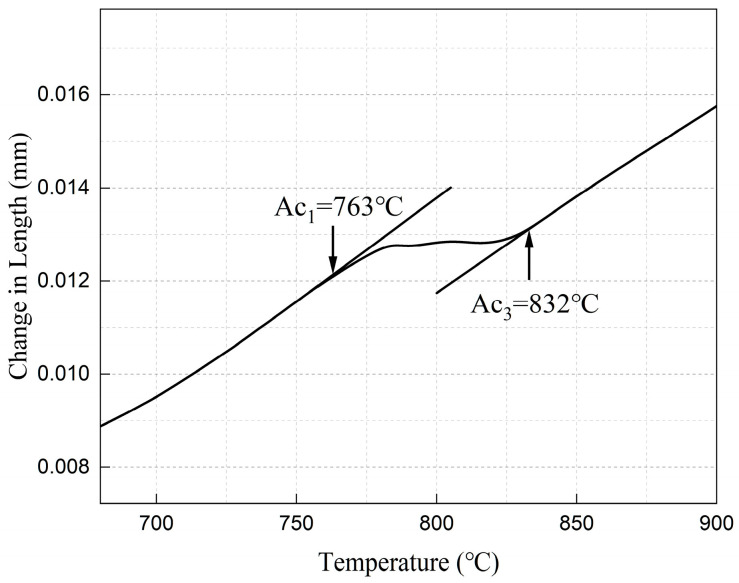
Thermal expansion curve of QT800-5 ductile iron.

The austenitization start (Ac_1_ = 763 °C) and finish (Ac_3_ = 832 °C) temperatures of the QT800-5 substrate were measured by NETZSCH DIL 402SE thermal dilatometer (NETZSCH-Gerätebau GmbH, Selb, Bavaria, Germany) (Figure 1). To avoid substrate phase transformation, annealing temperatures were set below Ac_1_ at three levels: 400 °C, 500 °C, and 600 °C. The Nb_0.75_ as-sprayed specimens were placed in a tube furnace (Tianjin Zhonghuan Electric Furnace Co., Ltd., Tianjin, China) and isothermally annealed for 2 h at each target temperature under an Ar atmosphere, with a heating rate of 10 °C·min^−1^ and furnace cooling to room temperature. The specimens are designated as-sprayed (HT0), HT400, HT500, and HT600. To verify that the designed annealing process does not induce detrimental microstructural changes in the QT800-5 substrate, uncoated QT800-5 substrates from the same batch and the substrates of the annealed coated specimens were simultaneously examined. The specimens were cold-mounted with epoxy resin (Shanghai Huifeng Resin Co., Ltd., Shanghai, China), progressively ground with 200 to 2000-grit SiC paper, and polished to a mirror finish using a diamond suspension. After etching with a 4% nital solution for 10–15 s, the metallographic structure of the substrates was examined using optical microscopy (OM, BX53M, Olympus Corporation, Tokyo, Japan) and scanning electron microscopy (SEM, TESCAN ORSAY HOLDING, a.s., Brno, Czech Republic), with a particular focus on the morphology of the graphite nodules and any matrix alterations.

### 2.3. Microstructural Characterization

Phase identification was conducted using a SmartLab 9 kW X-ray diffractometer (XRD, Rigaku Corporation, Tokyo, Japan) with Cu Kα radiation (40 kV, 40 mA). Scans were performed in continuous mode over a 2*θ* range of 20–80° at a rate of 10°·min^−1^; the step size was 0.02°. Phase indexing was performed with JADE 6.5 software (Materials Data Inc., Livermore, CA, USA).

Cross-sectional specimens were prepared by cold mounting, followed by sequential grinding with 200–2000-grit SiC paper and polishing with a 1.5 μm diamond suspension (Shanghai Yubo Mechanical & Electrical Technology Co., Ltd., Shanghai, China) to achieve a mirror finish. Microstructural observation and elemental mapping were performed using a VEGA3 SEM equipped with an energy dispersive X-ray spectroscopy (EDS) detector (Oxford Instruments plc, Abingdon, Oxfordshire, UK). Specimens were sputter-coated with gold using an ion sputtering coater (SBC-12, Beijing KYKY Technology Co., Ltd., Beijing, China) to prevent charging artifacts during SEM examination. It should be noted that SEM can resolve μm-scale Laves phase morphology and distribution but cannot identify nanometric precipitates. For each phase region (FCC matrix, Laves phase, Nb-rich zone), 3–5 point analyses were performed in randomly selected representative locations, and the results were averaged. All EDS data reported are in atomic percent (at.%), normalized to 100% including oxygen. When the Cr/Nb stoichiometric ratio is discussed, the reported values were recalculated after excluding the oxygen content.

Quantitative image analysis of the Laves phase was performed on BSE-SEM cross-sectional micrographs using ImageJ 1.54f software (National Institutes of Health, Bethesda, MD, USA). For the *x* = 0.75 composition under each condition (HT0, HT400, HT500, HT600), five non-overlapping fields at 1000× magnification were analyzed, with no less than 200 Laves phase particles counted per field to ensure statistical reliability. The bright-white Laves phase regions were segmented by threshold binarization, and the area fraction (*A_f_*), average equivalent circle diameter (*d_avg_*), and mean inter-precipitate spacing (*λ*) were determined. The inter-precipitate spacing was calculated as *λ* = *d_avg_* × [(1/*V_f_*)^1/2^ − 1], where *V_f_* is the volume fraction approximated by the measured area fraction.

### 2.4. Mechanical and Tribological Testing

Vickers microhardness was measured along the cross-sectional depth by HVSA-1000A hardness tester (Laizhou Huayin Test Instrument Co., Ltd., Laizhou, China), with a test load of 0.2 kgf, dwell time of 10 s, and indentation spacing of 50 μm. Ten indentations per specimen were averaged. Nanoindentation tests on specific phase regions in the coating cross-section were performed using a nanoindenter (KLA Instruments, Nano-mechanics Inc., Milpitas, CA, USA) at a maximum load of 50 mN, a loading rate of 100 nm·s^−1^, and a dwell time of 15 s. A minimum of five indents were made in each identifiable phase region (FCC matrix, Laves phase, and Nb-rich zone) in the CoCrFeNiNb_1.00_ coating cross-section. Phase identification was based on BSE contrast and confirmed by EDS spot analysis on adjacent areas prior to indentation.

Dry sliding tribological tests were conducted at room temperature using a reciprocating friction-and-wear tester (Rtec Instruments, San Jose, CA, USA) with a 6 mm-diameter tungsten carbide ball (Zhuzhou Cemented Carbide Group Co., Ltd., Zhuzhou, China) as the counterbody. Each test was run at a normal load of 20 N, 1 Hz frequency, 3 mm stroke, for 30 min. Each tribological test was repeated three times, and the reported values represent the average. Tests were conducted at an ambient temperature of 25 ± 2 °C and relative humidity of 40–60%. Post-test wear track profiles were acquired with a Bruker 3D optical profilometer (Bruker Nano GmbH, Karlsruhe, Germany); depth, width, and cross-sectional area were extracted from the profiles to calculate volumetric wear rate. The volumetric wear rate *W* was calculated using the equation *W* = *V*/(*F*·*L*), where *V* is the wear volume obtained from the cross-sectional area of the wear track (measured by profilometry) multiplied by the track length, *F* is the normal load, and *L* is the total sliding distance. Worn surfaces were then examined by SEM and EDS to characterize the operative wear mechanisms.

The initial Hertzian contact pressure was calculated using the classical elastic contact solution for a ball-on-flat geometry. For a 6 mm-diameter WC ball *E* = 620 GPa, *ν* = 0.24 pressed against the coating surface (*E* ≈ 200 GPa, *ν* ≈ 0.30 estimated for the HEA coating) at a normal load of 20 N, the maximum Hertzian contact pressure *P_max_* is approximately 2.87 GPa, and the contact radius a ≈ 49 μm. The corresponding mean contact pressure is approximately 1.91 GPa. The linear sliding speed at 1 Hz frequency and 3 mm stroke is 6 mm/s. Prior to tribological testing, the surface roughness of all polished coating specimens was measured using the Bruker 3D optical profilometer, yielding *Ra* values of 0.18 ± 0.03 μm (as-sprayed polished), 0.15 ± 0.02 μm (HT400), 0.14 ± 0.02 μm (HT500), and 0.16 ± 0.03 μm (HT600), confirming comparable initial surface conditions across all specimens.

To confirm that steady-state wear was reached for all tested conditions, the friction coefficient curves were analyzed as follows: the running-in period was defined as the initial interval during which the friction coefficient exhibits a monotonic increase or large-amplitude fluctuation, and the steady-state regime was identified as the subsequent period where the friction coefficient stabilizes within ±5% of the running average. For HT0 and HT400, steady state was reached at approximately 8–10 min; for HT500 and HT600, at approximately 4–6 min. The HT400 friction trace shows higher variability in the steady-state regime (standard deviation of the friction coefficient, *σ* = 0.038) compared with HT500 (*σ* = 0.012), which is attributed to intermittent pore-related debris generation rather than failure to reach steady state. The 30 min test duration was therefore sufficient for all conditions to develop a stabilized wear regime.

## 3. Results

### 3.1. Phase Composition and Microstructure of Coatings with Different Nb Contents

The bulk chemical composition of the as-sprayed CoCrFeNiNb*_x_* coatings was characterized by large-area EDS mapping, and the nominal and measured compositions are summarized in Table 1. The measured Nb content of all coatings is slightly lower than the nominal design value, with the Nb retention rate ranging from 79.0% to 85.7%.

This composition deviation is attributed to the differential deposition efficiency of elemental Nb powder during APS deposition. The melting point of elemental Nb (~2477 °C) is substantially higher than that of the pre-alloyed CoCrFeNi powder (~1390 °C). Under the same plasma conditions, partially high-melting-point Nb particles cannot be completely melted in-flight. The semi-molten or unmolten Nb particles have insufficient kinetic energy to adhere to the substrate and are more likely to rebound or be blown away by the plasma jet, resulting in a lower deposition efficiency of Nb compared with the CoCrFeNi matrix elements. The Nb retention rate decreases marginally with the increase in nominal Nb content, which is consistent with the increased proportion of unmolten Nb particles at higher Nb addition levels.

For compositional reproducibility, the standard deviation of the measured Nb content is controlled within ±1.4 at.% for all coatings, and the relative deviation of the matrix element (Co, Cr, Fe, Ni) content is less than 3% between different test fields. This indicates that, despite the differential deposition efficiency of Nb, the optimized APS parameters can still ensure good compositional uniformity and reproducibility in the coatings, which is sufficient to support subsequent microstructural and performance analyses.

**Figure 2:**
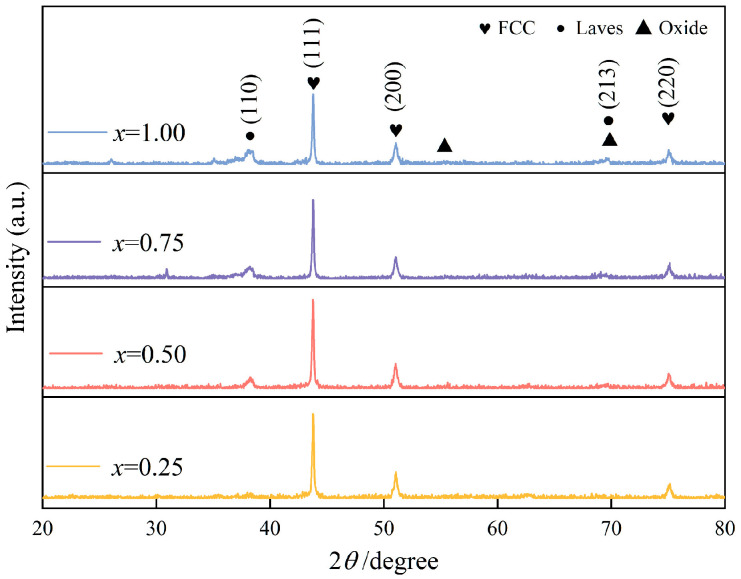
XRD patterns of the CoCrFeNiNb*_x_* high-entropy alloy coatings.

XRD patterns of the CoCrFeNiNb*_x_* coatings are shown in Figure 2. The *x* = 0.25 coating is single-phase FCC, with no secondary phase peaks discernible above the detection limit. Raising Nb to *x* = 0.50 introduces weak but identifiable Laves phase reflections at 2*θ* = 38.49°, 55.57°, and 69.63°. At *x* = 0.75 and 1.00, these reflections intensify while the FCC (111) peak undergoes a modest reduction in intensity. Jade 6.5 phase matching identifies the precipitate as the Cr_2_Nb-type Laves phase.

**Figure 3:**
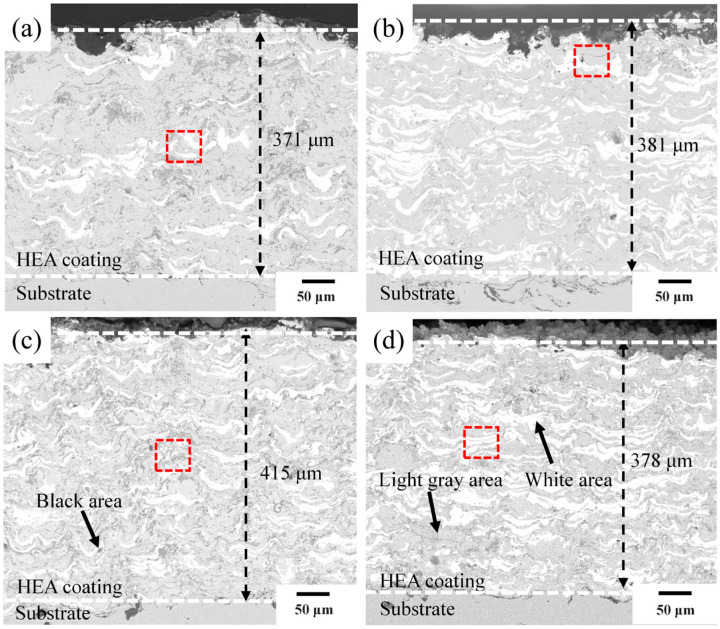
Cross-sectional morphologies of CoCrFeNiNb*_x_* high-entropy alloy coating: (**a**) Nb_0.25_, (**b**) Nb_0.50_, (**c**) Nb_0.75_, and (**d**) Nb_1.00_. The red boxes in each subfigure mark the representative regions selected for the subsequent high-magnification SEM observation, EDS elemental mapping and compositional point analysis.

Figure 3 shows cross-sectional SEM images of the CoCrFeNiNb*_x_* coatings. All coatings exhibit a compact, well-defined interface with the QT800-5 substrate and no visible microcracks or delamination. The coating thicknesses are *x* = 0.25 (371 μm), *x* = 0.50 (381 μm), *x* = 0.75 (415 μm), and *x* = 1.00 (378 μm). The interiors display a typical multi-phase heterogeneous structure with light-gray, dark-gray, bright-white, and black regions, representing the FCC matrix, Laves-phase-enriched zones, Nb-rich regions, and pores or oxide inclusions, respectively. The number of white Nb-rich domains and dark-gray Laves phase regions increases with higher Nb content.

**Figure 4:**
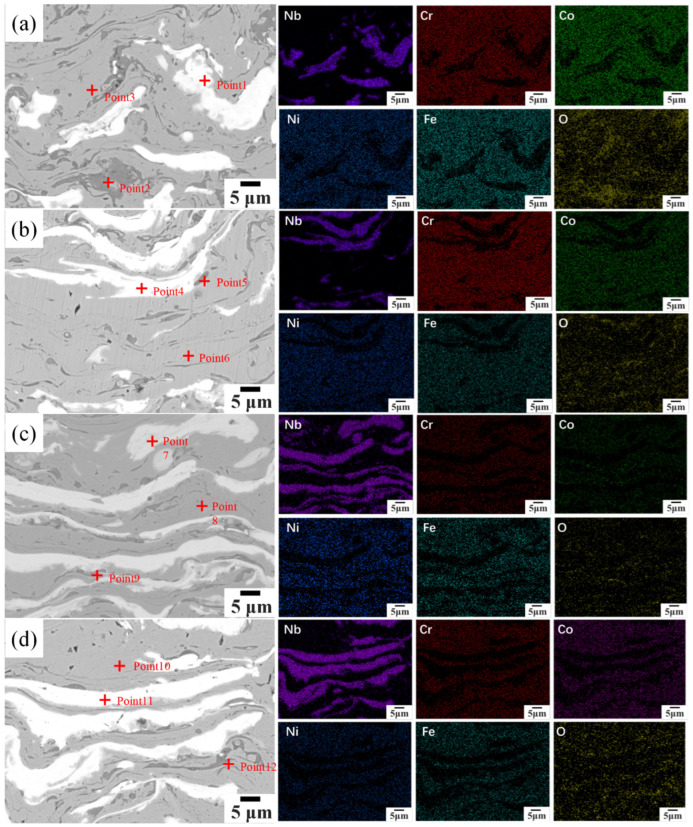
Scanning images and element distribution of the enlarged area of CoCrFeNiNb*_x_* high-entropy alloy coating: (**a**) Nb_0.25_, (**b**) Nb_0.50_, (**c**) Nb_0.75_, and (**d**) Nb_1.00_.

Figure 4 displays higher-magnification SEM images along with corresponding EDS elemental maps of selected areas. Table 2 summarizes EDS point analysis results from the labeled regions. At *x* = 0.25, a primary Nb-rich area with 64.3 at.% Nb (Point 1) coexists with niobium oxide inclusions (Point 2, O: 43.6 at.%) and a clean FCC solid solution region with a near-equiatomic distribution of principal elements (Point 3, O: 1.5 at.%). At *x* = 0.50 and 0.75, regions showing Cr/Nb atomic ratios (corrected for oxygen content) consistent with the 2:1 stoichiometry of Cr_2_Nb Laves phase are observed (Points 5 and 9: Cr31.8–36.9 at.%, Nb approximately 14 at.%), confirming Cr_2_Nb-type Laves phase precipitation. The O content in the FCC matrix increases to 10.0 at.% at *x* = 0.75 (Point 8). This oxygen incorporation originates from in-flight oxidation of the powder during APS, which affects the mechanical stability of the FCC matrix during sliding wear. At *x* = 1.00, a composite niobium oxide phase (Point 11: Nb 43.4 at.%, O 36.3 at.%) and oxygen-doped Laves phase regions (Point 12: O 24.4 at.%) are present.

EDS elemental maps reveal that the Nb signal is concentrated in specific clusters, in clear contrast to the spatial distributions of Cr and Fe. Co, Cr, Fe, and Ni are evenly spread within the FCC matrix, while the Cr signal is locally concentrated in Laves phase areas. Oxygen distribution closely matches the Nb-active zones. As Nb content rises, the variation range of main elements in the FCC matrix broadens from ±1.5 at.% to ±3.0 at.%.

### 3.2. Mechanical and Tribological Properties of Coatings with Different Nb Contents

**Figure 5:**
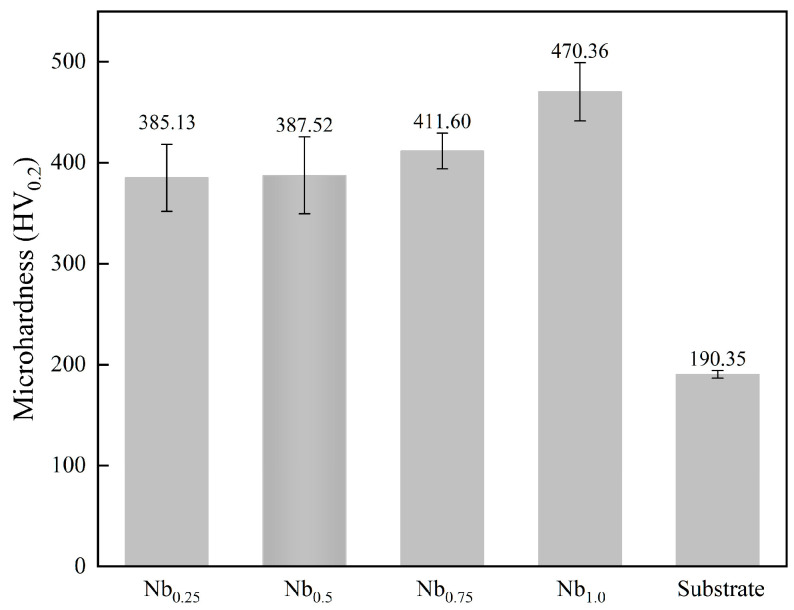
Microhardness of CoCrFeNiNb*_x_* high-entropy alloy coating.

Figure 5 shows the Vickers microhardness of the CoCrFeNiNb*_x_* coatings in relation to Nb content. The QT800-5 substrate has a hardness of 190.35 HV. All coatings exceed the substrate hardness significantly, and the hardness consistently rises with increasing Nb content: 385.13, 387.52, 411.60, and 470.36 HV at *x* = 0.25, 0.50, 0.75, and 1.00, respectively, representing a total increase of 22.1% across this range.

**Figure 6:**
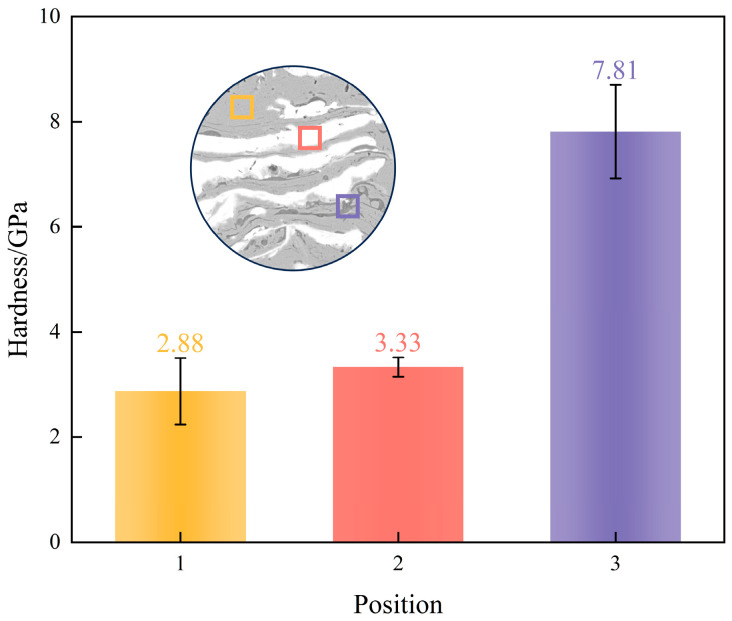
Nanoindentation hardness of distinct microstructural regions in the CoCrFeNiNb_1.00_ coating. The yellow, red, and purple columns, along with the corresponding colored indentation marks in the inset, correspond to the hardness of the FCC matrix, Nb-rich zone, and Laves phase, respectively.

Nanoindentation measurements across distinct microstructural regions of the CoCrFeNiNb_1.00_ coating are summarized in Figure 6. The dark-gray Laves phase returned a nanohardness of 7.81 GPa, compared with 3.33 GPa for the bright-white Nb-rich regions and 2.88 GPa for the light-gray FCC matrix. At roughly 2.7 times the hardness of the FCC matrix, the Cr_2_Nb-type Laves phase is clearly the dominant hard constituent in this coating.

**Figure 7:**
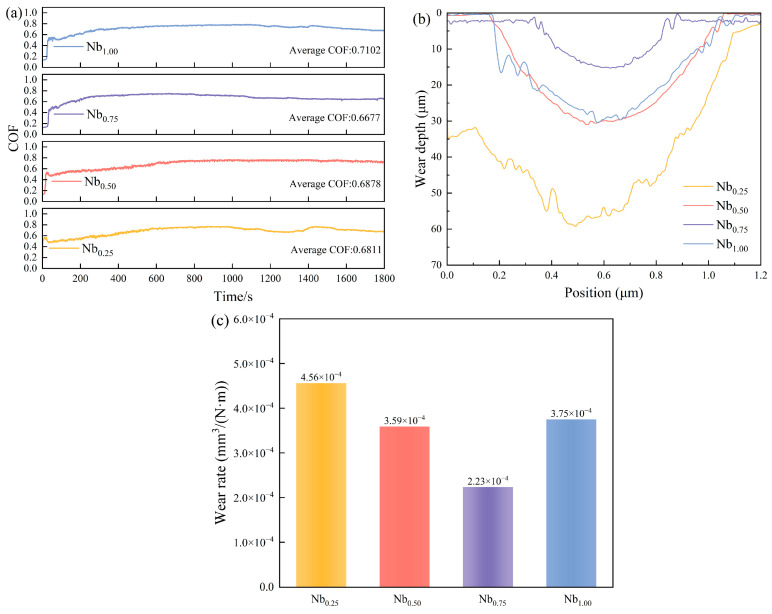
Tribological properties of CoCrFeNiNb*_x_* coatings: (**a**) friction coefficient vs. time; (**b**) wear scar cross-sectional depth profile; (**c**) volumetric wear rate.

Friction coefficient–time curves, wear track depth profiles, and volumetric wear rates are compiled in Figure 7. Among all compositions, *x* = 0.75 returned the lowest average friction coefficient (0.6677) and the most stable steady-state response, with little run-to-run scatter once sliding equilibrated. By contrast, *x* = 0.25 showed pronounced friction oscillations throughout, and *x* = 1.00 settled at a notably higher average of 0.7102. Wear track depths tell a consistent story: *x* = 0.75 reached only ~14 μm, whereas *x* = 0.25 reached ~59 μm, and *x* = 0.50 and 1.00 reached ~29 μm and ~30 μm, respectively. The volumetric wear rate mirrors this ranking, with *x* = 0.75 corresponding to the minimum of 2.23 × 10^−4^ mm^3^·N^−1^·m^−1^.

**Figure 8:**
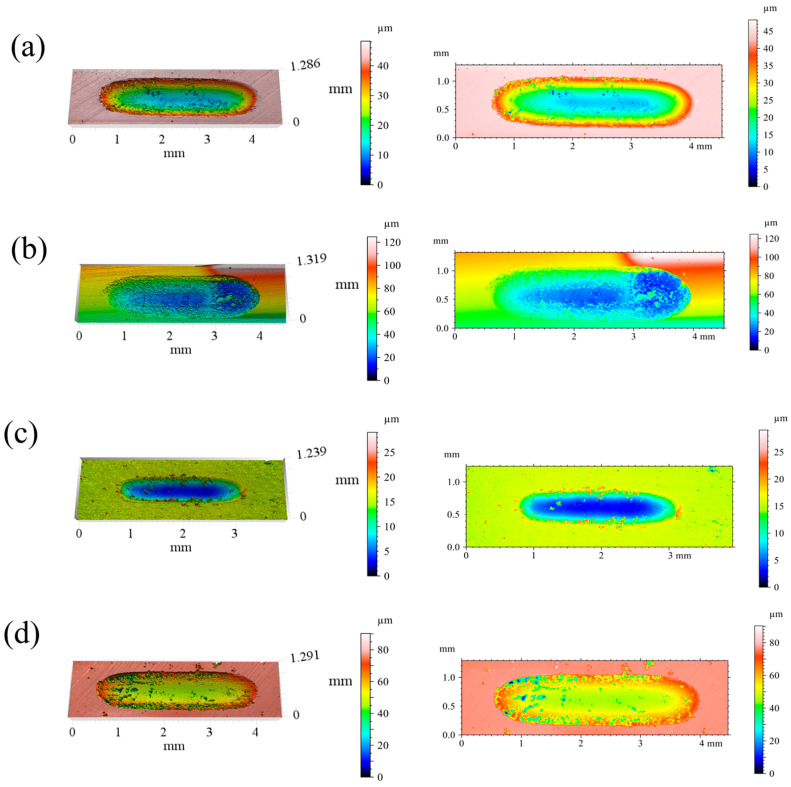
3D white-light interferometric profiles of wear tracks for CoCrFeNiNb*_x_* coatings: (**a**) *x* = 0.25, (**b**) *x* = 0.50, (**c**) *x* = 0.75, and (**d**) *x* = 1.00.

3D white-light interferometric profiles of the wear tracks are given in Figure 8. At *x* = 0.25, the worn surface is riddled with deep, through-penetrating pits characteristic of severe fatigue spalling. The *x* = 0.50 coating presents the roughest topography overall, with closely spaced isolated craters distributed across the track. Moving to *x* = 0.75, spalling pits are fewer and more confined, and the overall surface damage is markedly reduced. At *x* = 1.00, the track is dominated by a fine, uniform scratch pattern with no large-scale spalling evident.

**Figure 9:**
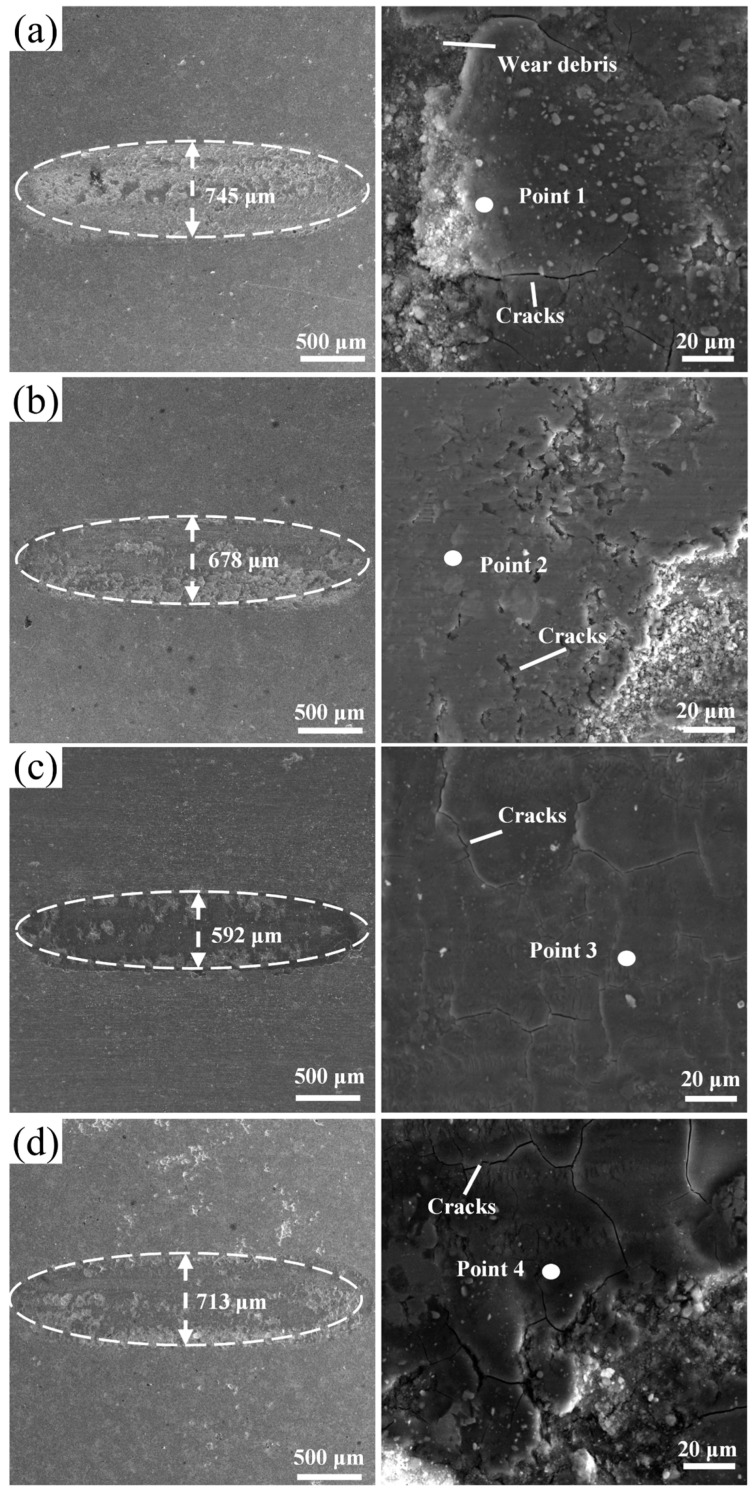
SEM images of the surface wear tracks of CoCrFeNiNb*_x_* coatings (**a**) Nb_0.25_, (**b**) Nb_0.50_, (**c**) Nb_0.75_, and (**d**) Nb_1.00_.

Figure 9 shows SEM micrographs of the worn surfaces; Table 3 lists EDS compositions of the marked regions. Wear track widths are ranked as *x* = 0.75 (592 μm) < *x* = 0.50 (678 μm) < *x* = 1.00 (713 μm) < *x* = 0.25 (745 μm). The *x* = 0.25 coating wear track displays plowing tears along its edges, numerous transverse microcracks, and platelet wear debris, with a surface O content of 65.5 at.%. The *x* = 0.50 coating shows distinct transverse grooves and large-area spalling pits (O: 51.3 at.%). The *x* = 0.75 coating surface features stripe-like damage parallel to the sliding direction, with radiated crack branches and fine particulate debris (O: 65.0 at.%). The *x* = 1.00 coating has smooth track edges and only sparse, short microcracks at high magnification, with the lowest surface O content (45.5 at.%).

### 3.3. Thermal Properties of QT800-5 and Phase Composition of the Annealed Nb_0.75_ Coating

**Figure 10:**
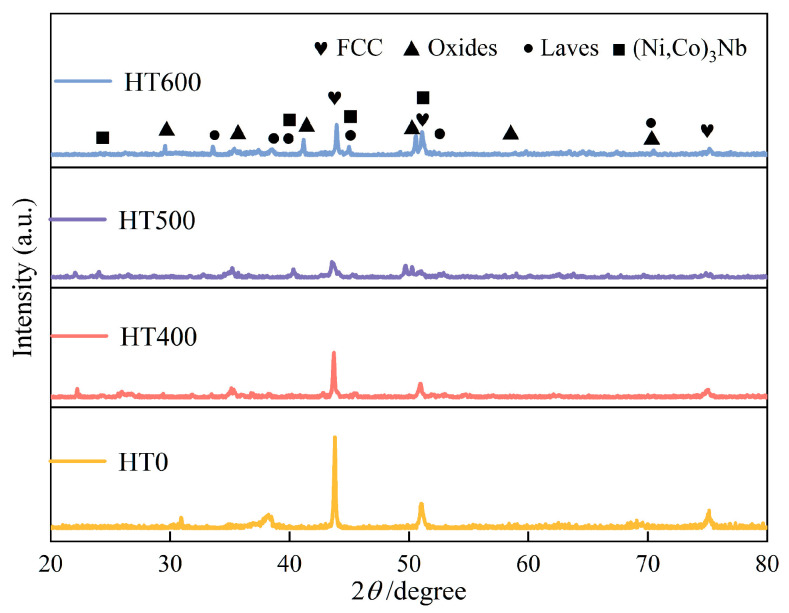
XRD patterns of CoCrFeNiNb_0.75_ high-entropy alloy coating after annealing.

Figure 10 displays the XRD patterns of the Nb_0.75_ coating in its as-sprayed state (HT0) and after annealing at 400 °C, 500 °C, and 600 °C. For the HT400 coating, the intensity of the main FCC phase (111) peak decreases, and weak oxide peaks along with Cr_2_Nb-type Laves phase characteristic peaks increase. The HT500 coating is primarily composed of FCC and Laves dual phases. For the HT600 coating, the peaks associated with the Laves phase increase further in intensity, while peaks of the (Ni,Co)_3_Nb phase appear and reach their maximum. At the same time, the intensity of the FCC phase diffraction peaks shows an unusual increase.

### 3.4. Microstructure of the Annealed Nb_0.75_ Coating

**Figure 11:**
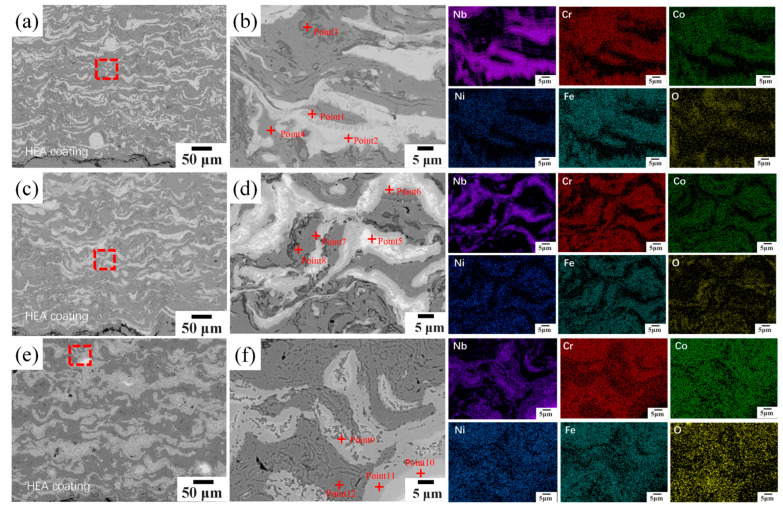
Cross-sectional morphology of CoCrFeNiNb_0.75_ coating after different temperature annealing: (**a**,**b**) HT400, (**c**,**d**) HT500, and (**e**,**f**) HT600. The red boxes in (**a**,**c**,**e**) mark the representative regions selected for the high-magnification observation and EDS compositional characterization shown in (**b**,**d**,**f**), respectively.

Figure 11 shows cross-sectional SEM images of the Nb_0.75_ coating after annealing at 400 °C, 500 °C, and 600 °C; Table 4 summarizes EDS point analysis results.

The HT400 coating maintains the typical lamellar as-sprayed structure and contains numerous irregular fine blocky precipitates. Point 1 displays a pronounced enrichment of Nb (24.7 at.%), Cr (20.0 at.%), and Fe (22.0 at.%), consistent with the stoichiometry of the Cr_2_Nb-type Laves phase, indicating the beginning of Laves phase precipitation.

In the HT500 coating, light-gray elongated Laves-phase precipitates are observed along grain boundaries (Point 6: Nb 20.3 at.%, Co 26.0 at.%, Ni 29.3 at.%), with the Laves-phase distribution appearing more uniform and the microstructure denser.

In addition, local (Ni,Co)_3_Nb intermetallic phase was identified by EDS point analysis in the HT500 coating (Point 6: Nb 20.3 at.%, Co 26.0 at.%, Ni 29.3 at.%), matching the 3:1 stoichiometric ratio of (Ni,Co) to Nb within the typical ±2–3 at.% accuracy of EDS. However, no corresponding diffraction peak of (Ni,Co)_3_Nb phase was observed in the XRD pattern of the HT500 sample. This discrepancy is attributed to the extremely low volume fraction and fine nanoscale distribution of (Ni,Co)_3_Nb precipitates, which are below the XRD detection limit (typically ~2–5 vol.%). Such fine intermetallic precipitates can be readily captured by high-resolution local EDS analysis but are too sparse and fine to produce detectable diffraction signals, which is a common phenomenon in low-temperature annealed high-entropy alloys.

The HT600 coating shows significant microstructural homogenization, with precipitate morphology changing from elongated to nearly spherical. Point 9 corresponds to Cr_2_Nb-type Laves phase (Nb 23.6 at.%, Cr 20.2 at.%, Fe 18.3 at.%), while Point 11 identifies a (Ni,Co)_3_Nb intermetallic phase (Nb 26.9 at.%, Co 21.4 at.%, Ni 21.1 at.%) with negligible oxygen content.

The quantitative statistical results of the Cr_2_Nb-type Laves phase are summarized in Table 5. For the as-sprayed HT0 coating, the Laves phase exists as coarse primary precipitates with an average size of 3.98 ± 0.18 μm, an area fraction of 10.79 ± 0.32%, and a mean inter-precipitate spacing of 5.23 ± 1.12 μm. After annealing at 400 °C (HT400), the diffusion of Nb atoms is limited at low temperature, so only a small number of fine secondary Laves phase precipitates are formed. As a result, the average size of Laves phase slightly decreases to 3.94 ± 0.15 μm, the area fraction marginally increases to 10.87 ± 0.28%, and the inter-precipitate spacing remains almost unchanged at 5.18 ± 1.08 μm.

When the annealing temperature rises to 500 °C (HT500), the diffusion rate of Nb atoms in the FCC matrix is significantly accelerated, which promotes the continuous nucleation and precipitation of a large number of fine secondary Laves phase. Consequently, the area fraction of Laves phase increases sharply to 16.03 ± 0.45%, the average size increases to 4.37 ± 0.21 μm, and the inter-precipitate spacing decreases significantly to 3.82 ± 0.91 μm. The dense distribution of Laves phase with fine size and small spacing greatly enhances the Orowan strengthening effect, which is the main reason for the significant improvement of the microhardness and wear resistance of the coating at this annealing temperature.

As the annealing temperature further increases to 600 °C (HT600), the Laves phase undergoes continuous precipitation and slight Ostwald ripening: the fine secondary Laves phase particles continue to precipitate and partially merge into coarse particles, leading to a further increase in the average size to 4.50 ± 0.23 μm, a continuous rise in the area fraction to the peak value of 18.65 ± 0.52%, and a further reduction in the inter-precipitate spacing to 3.56 ± 0.88 μm. The high area fraction and dense distribution of the Laves phase maintain the coating’s excellent mechanical properties at high annealing temperatures, and the Orowan strengthening effect remains the dominant mechanism.

### 3.5. Mechanical and Tribological Properties of the Annealed Nb_0.75_ Coating

**Figure 12:**
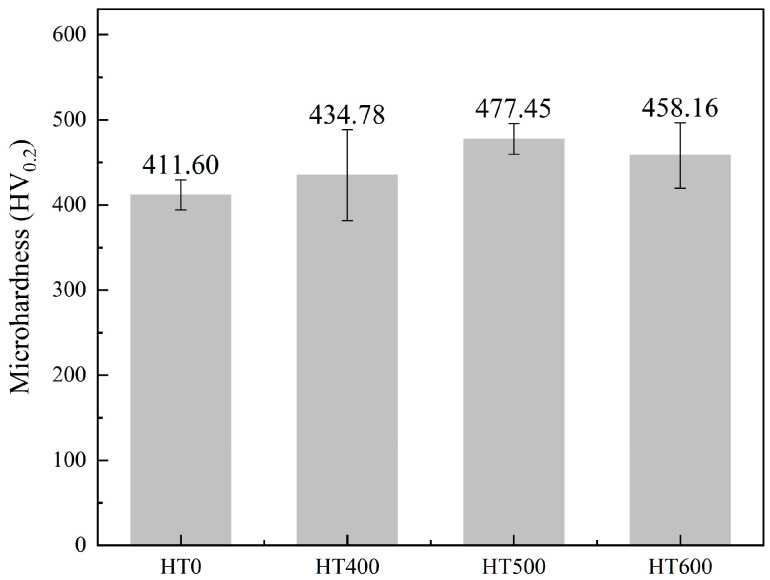
Microhardness of CoCrFeNiNb_0.75_ high-entropy alloy coating after annealing.

Figure 12 shows the Vickers microhardness of the Nb_0.75_ coating as a function of annealing temperature. It begins at 411.60 HV in the HT0 condition, rises to 434.78 HV at HT400, reaches a peak of 477.45 HV at HT500 (a 16% increase relative to HT0), and then slightly decreases to 458.16 HV at HT600, remaining well above the as-sprayed level.

**Figure 13:**
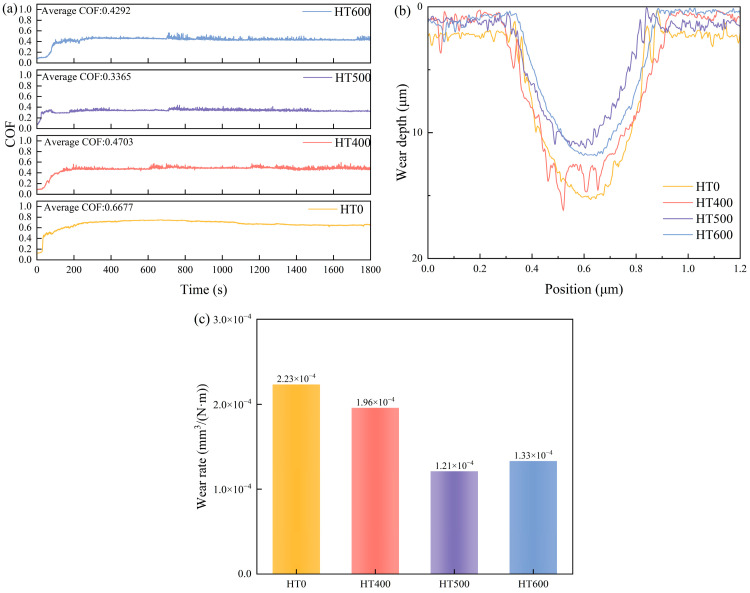
Tribological performance parameters of CoCrFeNiNb_0.75_ high-entropy alloy coating after heat treatment: (**a**) coefficient of friction, (**b**) wear scar depth, and (**c**) wear rate.

Figure 13 summarizes the tribological performance of the annealed Nb_0.75_ coatings. All heat-treated coatings exhibit significantly lower friction coefficients than HT0, and all show sawtooth-like friction traces linked to the periodic buildup and detachment of wear debris, as shown in Figure 13a. The HT500 coating reaches the steady-state phase most quickly and has the lowest average friction coefficient (0.3365), while HT400 has the highest (0.4703), and HT600 falls in between at 0.4292.

Wear track depth measurements show that HT500 has the shallowest wear track depth (~12 μm). The HT400 depth profile displays sharp spikes corresponding to residual as-sprayed micropore defects. The HT600 profile is relatively smooth, with a depth slightly greater than that of the HT500, as shown in Figure 13b. Volumetric wear rates follow the same pattern: HT500 has the lowest wear rate of 1.21 × 10^−4^ mm^3^·N^−1^·m^−1^, representing a 45% reduction compared to HT0; HT400 and HT600 have wear rates of 1.95 × 10^−4^ and 1.33 × 10^−4^ mm^3^·N^−1^·m^−1^, respectively, as shown in Figure 13c.

**Figure 14:**
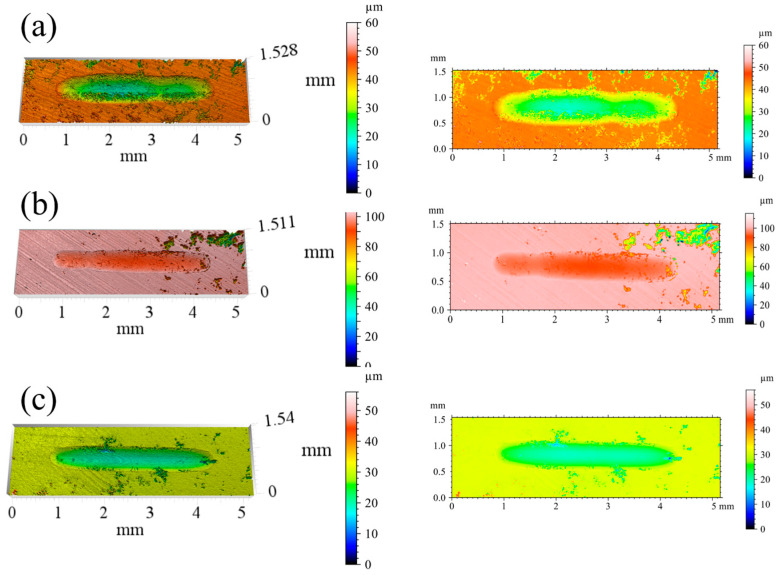
3D white-light interferometric wear track profiles of annealed coatings: (**a**) HT400, (**b**) HT500, and (**c**) HT600.

Figure 14 shows 3D white-light interferometric profiles of the worn surfaces. The HT500 coating has the shallowest depth gradient along the track edges (<5 μm·mm^−1^), the smoothest surface, and the best resistance to plowing. The HT600 coating exhibits a maximum wear depth of approximately 20 μm and a slightly wider track than HT500. The HT400 coating worn surface shows pronounced furrows and extensive spalling.

**Figure 15:**
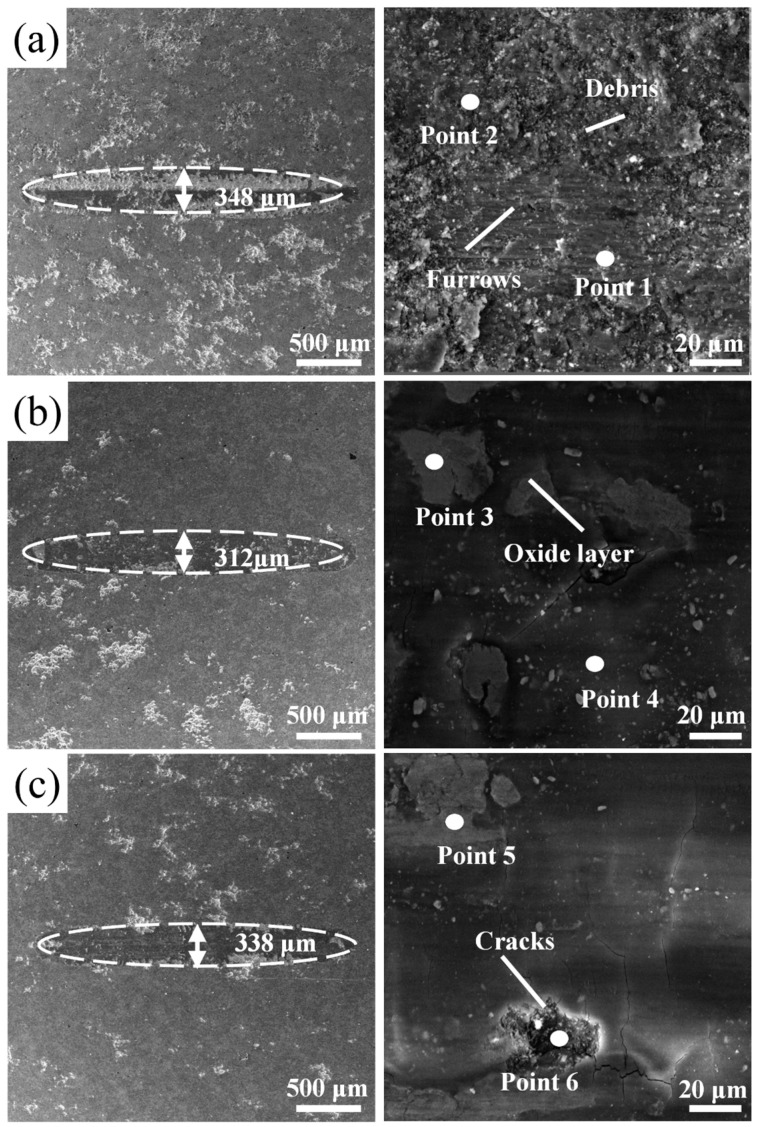
SEM micrographs of worn surfaces of annealed coatings: (**a**) HT400, (**b**) HT500, and (**c**) HT600.

SEM micrographs of worn surfaces for the annealed coatings are shown in Figure 15; EDS compositions are listed in Table 6. Wear track widths rank as HT500 (312 μm) < HT600 (338 μm) < HT400 (348 μm) < HT0 (592 μm). On the HT500 surface, scattered white oxide patches mark spalled regions; EDS at Point 3 (Nb: 50.4 at.%) confirms these as Laves phase oxidation zones, whereas the neighboring intact area (Point 4) carries considerably less oxygen (44.2 at.%), suggesting oxidation remained localized. HT400 exhibits mixed abrasive–adhesive damage: groove zones register O at 32.5 at.% (Point 1), and debris accumulation zones reach 64.1 at.% (Point 2), consistent with secondary oxidation of detached wear particles. HT600 develops pits of roughly 15–20 μm diameter; fine cracks run along grain boundaries at the pit edges, and O content within these cracks reaches 53.6 at.% (Point 6), pointing to pronounced grain boundary oxidation.

### 3.6. Effect of Annealing Treatment on the Microstructure of the QT800-5 Ductile Iron Substrate

**Figure 16:**
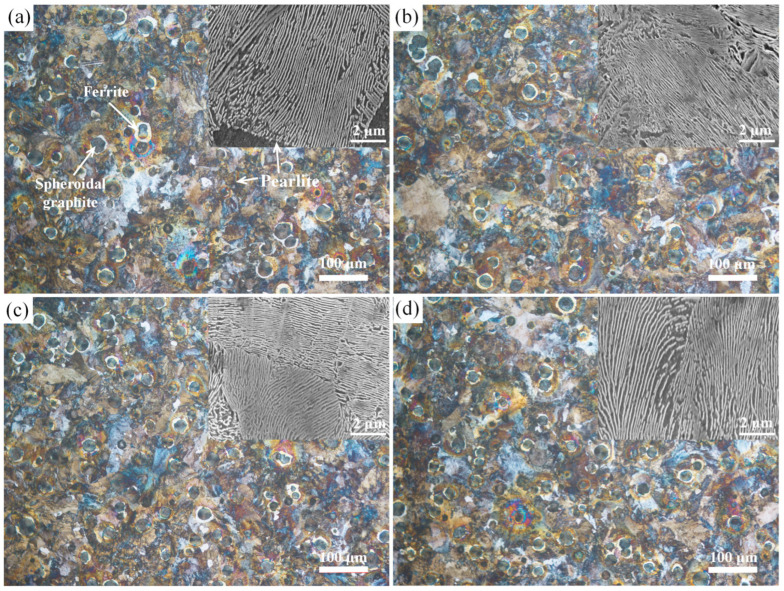
Optical micrographs and high-magnification SEM of the QT800-5 substrate: (**a**) as-received; (**b**) 400 °C anneal; (**c**) 500 °C anneal; (**d**) 600 °C anneal.

Figure 16 illustrates the metallographic microstructures of the QT800-5 ductile iron in its as-received state and after annealing at different temperatures. The as-received QT800-5 substrate exhibits the typical microstructural features of this grade: spheroidal graphite is uniformly distributed within a predominantly pearlitic matrix, free from obvious metallurgical defects such as flake graphite, shrinkage cavities, or inclusions. Annealing at 400, 500, and 600 °C introduced no detrimental microstructural changes. Graphite nodule morphology, size, and distribution were indistinguishable from the as-received condition, with no evidence of dissolution, loss of nodularity, or interfacial debonding. The pearlitic matrix likewise retained its lamellar character throughout, showing no signs of austenitization, spheroidization, grain coarsening, or thermal cracking.

High-magnification SEM of the pearlite lamellae (insets, Figure 16) was used to assess any subtler matrix changes. In the as-received condition, ferrite and cementite lamellae alternate regularly, with straight, well-defined phase boundaries and no spheroidization or network fragmentation. After annealing at all three temperatures, this lamellar arrangement was fully preserved: boundaries remained sharp, and neither cementite fracture, spheroidization, nor coarsening was detected.

Pearlite inter-lamellar spacing measurements are tabulated in Table 7. The as-received matrix had an average spacing of (164 ± 12) nm; values after annealing at 400, 500, and 600 °C were (169 ± 8) nm, (167 ± 11) nm, and (174 ± 17) nm, respectively. One-way ANOVA found no significant difference across these conditions (*p* > 0.05), indicating that the low-temperature annealing leaves the fine pearlitic structure intact and the bulk strength and toughness of the substrate unaffected.

This stability is straightforwardly explained by the temperature window chosen: all three annealing temperatures fall well below the Ac_1_ of QT800-5 (763 °C), keeping the substrate clear of any austenitization-driven transformation. Taken together, the data confirm that low-temperature annealing in this range is compatible with ductile iron and poses no risk to the substrate’s metallurgical integrity when used as a post-treatment for HEA coatings. The substrate hardness values showed no statistically significant change after annealing, with only a minor, gradual reduction observed at the highest annealing temperatures, further confirming that the designed low-temperature annealing protocol preserves the substrate’s intrinsic mechanical properties.

## 4. Discussion

### 4.1. Effect of Nb Content on Phase Composition and Microstructure

The phase evolution from single FCC at *x* = 0.25 to a dual FCC + Cr_2_Nb-type Laves structure at higher Nb contents is governed by two competing factors: the solubility limit of the FCC lattice and the large atomic size mismatch of Nb. At *x* = 0.25, Nb sits within the solubility limit and is incorporated as a substitutional solute, which is why no secondary phase reflections appear in the XRD pattern. Beyond *x* = 0.50, the elastic strain energy associated with excess Nb becomes thermodynamically untenable, and Nb preferentially pairs with Cr to nucleate Cr_2_Nb-type Laves phase at grain boundaries and inter-lamellar interfaces [23]. The steady rise in Laves phase peak intensity in XRD and in the areal fraction of dark-gray regions in SEM cross-sections with increasing *x* shows that both nucleation rate and volume fraction scale directly with Nb addition.

It should be noted that the above phase evolution analysis is based on the specific APS parameters and annealing conditions employed in this study. The thermodynamic driving force for Cr_2_Nb-type Laves phase formation derives from the strong negative mixing enthalpy between Nb and the transition metal matrix elements (*ΔH_mix_* < 0) and the large atomic size mismatch (*δ* > 6.6%), which together render extended Nb solid solution thermodynamically unfavorable beyond *x* = 0.25.

The heterogeneous microstructure seen in all as-sprayed coatings traces back to the extreme thermal and kinetic conditions of APS. Nb has a high melting point (~2477 °C) and commonly passes through the plasma jet only partially molten; ultra-rapid solidification (~10^6^ K·s^−1^) then freezes in compositional inhomogeneity, leaving primary Nb-rich clusters throughout the coating. The high oxygen affinity of Nb under plasma conditions drives in-flight oxidation, explaining the strong spatial correlation between O and Nb in EDS maps. As Nb content rises, lattice distortion in the FCC matrix intensifies and the compositional fluctuation range of the principal elements widens from ±1.5 to ±3.0 at.%. The resulting strain energy provides additional thermodynamic driving force for desolubilization, promoting Laves phase nucleation and growth on annealing.

Image analysis (Image J, threshold segmentation on BSE-SEM images, 5 fields per specimen) estimates the oxide + pore area fraction as: 4.1 ± 0.8% (*x* = 0.25), 4.6 ± 1.1% (*x* = 0.50), 7.1 ± 0.9% (*x* = 0.75), and 7.8 ± 1.3% (*x* = 1.00). The increasing trend correlates with the higher oxygen affinity of Nb-rich regions at elevated Nb contents.

### 4.2. Mechanisms Governing the Effect of Nb Content on Mechanical and Tribological Properties

Hardness rises monotonically with Nb content, reflecting contributions from both solid solution and second-phase strengthening. Oversized Nb atoms distort the FCC lattice, generating dilatational stress fields that interact with gliding dislocations, raise the Peierls stress, and impede slip, producing measurable solid solution hardening even at *x* = 0.25. With more Nb, the Cr_2_Nb-type Laves phase precipitates in increasing amounts; as a topologically close-packed intermetallic with a complex crystal structure, it resists dislocation motion and effectively pins grain boundaries. Nanoindentation puts the Laves phase hardness at ~7.81 GPa, roughly 2.7 times that of the FCC matrix (~2.88 GPa), consistent with values reported elsewhere [24]. As the Laves phase volume fraction grows with Nb addition, coating hardness climbs from 385.13 HV at *x* = 0.25 to 470.36 HV at *x* = 1.00, about 2.47 times the substrate hardness of 190.35 HV.

Unlike hardness, the wear rate does not decrease monotonically with Nb content; instead, a clear optimum exists at *x* = 0.75, reflecting the competing demands of hardness and toughness during sliding contact. Without a hard Laves phase network (*x* = 0.25), the coating deforms plastically under cyclic contact; frictional heating accelerates oxide film growth, and the brittle oxide layer periodically fractures and delaminates, producing oxidative delamination wear and the highest measured wear rate. At *x* = 0.50, the Laves phase is too sparsely distributed to form a load-bearing network, leaving the soft FCC matrix exposed to adhesive transfer under shear and prone to block-type spalling and mixed adhesive–oxidative damage. At *x* = 0.75, a sufficient volume of uniformly dispersed Laves particles is present: the hard phase resists abrasive cutting and dislocation glide, while the FCC matrix accommodates local stress concentrations plastically, blunting crack tips before they propagate. This combination produces the lowest wear rate (2.23 × 10^−4^ mm^3^·N^−1^·m^−1^) and the most stable friction trace. Pushing Nb further to *x* = 1.00 tips the balance toward brittleness: despite the highest hardness of any composition (470.36 HV), the wear rate exceeds that of *x* = 0.75.

Although direct fracture toughness measurements were not performed, several lines of indirect evidence support the proposed brittleness increase at *x* = 1.00. First, the nanoindentation results show that the Laves phase (7.81 GPa) is 2.7 times harder than the FCC matrix, and its volume fraction increases substantially from *x* = 0.75 to *x* = 1.00, reducing the toughening capacity of the ductile FCC matrix. Second, the worn surface of *x* = 1.00 exhibits fine, uniform scratch patterns without large-scale spalling (Figure 8d), which is characteristic of brittle micro-cutting rather than ductile deformation, consistent with a coating that is hard but prone to interfacial crack propagation. Third, the wear track width of *x* = 1.00 (713 μm) is larger than that of *x* = 0.75 (592 μm) despite higher hardness, suggesting that material removal at *x* = 1.00 proceeds through interfacial debonding between the overgrown Laves phase and the matrix rather than through bulk plastic deformation. The superior wear performance of *x* = 0.75 thus comes down to an effective balance between hard-phase reinforcement and matrix toughness.

### 4.3. Effect of Annealing at 400–600 °C on Phase Composition and Microstructural Evolution of the Nb_0.75_ Coating

The phase transformations observed during annealing of the Nb_0.75_ coating originate from the thermodynamic metastability imposed by APS processing. The ultra-rapid solidification rate (~10^6^ K·s^−1^) traps a large excess of Nb atoms within the FCC lattice, far beyond the equilibrium solubility limit, producing a highly strained, Nb-supersaturated solid solution. This metastable state carries substantial free energy, which acts as a driving force for precipitation on reheating. Annealing at 400–600 °C activates diffusion and nudges the system toward equilibrium; the resulting phase transformations and microstructural changes vary markedly with temperature, as discussed below.

Although the as-sprayed HT0 coating already contains some Laves phase, 400 °C is insufficient to drive significant diffusion. Nb atoms can reach high-energy sites such as grain boundaries and lattice defects, where they react with Cr and Fe to nucleate fine, blocky Cr_2_Nb-type Laves phase precipitates. XRD shows weak Laves phase peaks, and SEM reveals a small number of fine blocky precipitates. The coating still maintains the lamellar as-sprayed structure and some residual micropore defects, leading to poor microstructural uniformity. The decrease in FCC (111) peak intensity at 400 °C is attributed to the consumption of Cr and Nb from the supersaturated FCC matrix during Laves phase precipitation, which reduces the volume fraction of the FCC phase.

At 500 °C (HT500), within the tested temperature range of 400–600 °C, the desolubilization rate of Nb and the growth rate of the Laves phase achieve the best balance: abundant Laves phase precipitates uniformly without significant coarsening. XRD shows a clear increase in Laves phase peak intensities, and SEM reveals elongated Laves precipitates along grain boundaries with improved coating density and less microporosity. However, the characteristic composition of (Ni,Co)_3_Nb was detected at Point 6 of HT500 in Table 4 (Co 26.0 at.%, Ni 29.3 at.%, Nb 20.3 at.%, (Co + Ni)/Nb ≈ 2.72, close to 3:1), suggesting that a small amount of (Ni,Co)_3_Nb phase not detected by XRD was precipitated out.

At 600 °C (HT600), long-range atomic diffusion is activated, leading to two simultaneous effects. First, the previously precipitated Laves phase coarsens, as shown by narrower XRD peak widths and a change in shape from elongated to nearly spherical precipitates in SEM images. Second, Nb atoms move and combine with Ni/Co atoms to form the more thermodynamically stable (Ni,Co)_3_Nb ordered intermetallic compound, whose XRD peaks reach maximum intensity at this temperature. The increase in FCC diffraction peak intensity at 600 °C is counterintuitive, as precipitation should deplete the FCC phase. Several factors may contribute to this observation: (i) recovery of the FCC matrix at 600 °C releases accumulated lattice strain, reducing peak broadening and increasing apparent intensity; (ii) microstructural densification reduces X-ray absorption by porosity, increasing the effective diffracting volume; and (iii) compositional homogenization of the remaining FCC matrix may sharpen diffraction peaks. However, distinguishing among these contributions requires additional characterization (e.g., Williamson–Hall analysis for strain–size deconvolution), which is beyond the scope of the present study. We will conduct in-depth characterization to verify this phenomenon in our follow-up research.

Furthermore, quantitative statistical results of the inter-lamellar spacing, combined with high-magnification SEM observations, confirm that annealing at 400–600 °C does not induce spheroidization, coarsening, or degradation of the pearlite in the ductile iron substrate, even at the micrometer-scale fine-structure level. Since the pearlite lamellar spacing is the critical microstructural parameter dictating the strength, hardness, and toughness of QT800-5 ductile iron, this stability directly proves that the designed low-temperature annealing process completely preserves the intrinsic mechanical properties of the substrate. This fundamentally resolves the industry pain point of the mismatch between conventional coating post-treatments and the thermal sensitivity of ductile iron.

It should be acknowledged that the current characterization is limited to XRD, SEM, and EDS, which cannot resolve nanometric precipitates or provide crystallographic orientation information. EBSD and TEM analysis of the Nb_0.75_–HT500 condition is planned as future work to directly verify nanoscale precipitation and texture evolution. Furthermore, it should be explicitly noted that residual stress magnitudes in the as-sprayed and annealed coatings were not experimentally determined in this study. While residual tensile stresses are widely reported in APS coatings and their relief during annealing is thermodynamically expected, the present work does not include direct measurements (e.g., via the XRD sin^2^ψ method or substrate curvature analysis). Accordingly, all discussion passages attributing crack initiation or tribological degradation to inferred residual tensile stresses should be understood as inferred mechanisms based on established understanding of APS coating behavior, not as conclusions supported by independent stress measurement data from this study. Residual stress characterization by the sin^2^ψ method is planned as future work to quantitatively validate these inferences.

### 4.4. Effect of Annealing Temperature on Mechanical Properties of the Nb_0.75_ Coating

Hardness in the *x* = 0.75 coating rises from 411.60 HV (HT0) to a peak of 477.45 HV (HT500), then eases back to 458.16 HV (HT600), tracing the sequence of Laves phase nucleation, growth, and coarsening. The non-monotonic trend reflects a shifting balance among precipitation strengthening, solid solution depletion, and microstructural densification.

At 400 °C, thermal energy is enough only for short-range Nb diffusion to grain boundaries and lattice defects, where fine blocky Laves phase nucleates and begins to pin dislocations. Partial stress relief and a modest reduction in microporosity add further hardening, bringing hardness to 434.78 HV. Long-range diffusion remains restricted at this temperature, so the Laves phase is limited in both amount and spatial uniformity, and the lamellar as-sprayed structure with residual micropores is largely intact.

At 500 °C, diffusion kinetics reach a window where Nb desolubilization and Laves phase growth are optimally balanced. Precipitates are numerous and uniformly distributed before any coarsening sets in, maximizing phase boundary density and dislocation-pinning sites. Simultaneous closure of inter-lamellar micropores densifies the coating further, and together these effects drive hardness to its peak of 477.45 HV-a 16% increase over the as-sprayed condition.

At 600 °C, long-range diffusion triggers Ostwald ripening: precipitates coarsen from elongated to near-spherical, phase boundary area shrinks, and dislocation-pinning efficiency drops, partially eroding the precipitation-strengthening gain. FCC matrix recovery releases lattice strain, and the newly formed (Ni,Co)_3_Nb intermetallic offers some compensating hardening, but neither effect is large enough to offset the coarsening loss. Hardness settles at 458.16 HV, still well above the as-sprayed baseline, meaning the precipitation reactions from lower-temperature annealing are not fully undone at 600 °C.

### 4.5. Effect of Annealing Temperature on Tribological Performance and Wear Mechanisms

The as-sprayed Nb_0.75_ coating contains inferred residual tensile stresses and inter-lamellar pore defects that act as preferential sites for crack initiation during sliding contact. Annealing at all three temperatures reduces these defects to varying degrees, and the resulting wear rate improvements reflect the interplay between precipitation strengthening, microstructural densification, and the strength–ductility balance of the coating. The 500 °C condition produces the most favorable outcome across all three factors simultaneously, as discussed below.

The HT500 coating achieves the best wear performance (wear rate: 1.21 × 10^−4^ mm^3^·N^−1^·m^−1^, 45% below HT0), with a wear mechanism of mild oxidative–fatigue wear. The uniformly dispersed Laves phase forms a stable hard-phase support network that effectively suppresses abrasive cutting and the initiation and propagation of subsurface cracks. Improved microstructural density minimizes defect-induced stress concentrations; the good plasticity of the FCC matrix accommodates local stress through plastic deformation, deflecting rather than propagating cracks. The self-repairing Nb-containing composite oxide film additionally suppresses oxidative wear. Together, these factors yield the shallowest wear track, narrowest track width, and lowest friction coefficient.

The relatively poor wear performance of HT400 stems from two unresolved microstructural liabilities. First, the sparse and non-uniform Laves phase distribution at 400 °C is insufficient to form a load-bearing hard-phase network, leaving the FCC matrix exposed to direct contact stress. Second, residual through-thickness micropore defects—largely intact at this low annealing temperature—act as stress concentrators under cyclic loading, triggering material spalling. Spalled fragments oxidize and re-enter the contact zone as abrasive debris, plowing the surface and sustaining mixed abrasive–oxidative wear. The combined effect of these two factors produces the highest average friction coefficient and a wear rate of 1.95 × 10^−4^ mm^3^·N^−1^·m^−1^ among the annealed specimens.

Annealing at 600 °C produces a microstructurally homogeneous coating free of significant porosity, yet Laves phase coarsening introduces a new vulnerability: the enlarged Laves–FCC interfaces have lower cohesive strength, and under cyclic frictional loading these interfaces preferentially debond, generating spalling craters ~15–20 μm in diameter. Oxygen infiltrates the resulting crack networks along grain and phase boundaries, as evidenced by the O content of 53.6 at.% measured inside boundary cracks by EDS, establishing a mixed adhesive–oxidative wear mode. Notably, the newly formed (Ni,Co)_3_Nb phase provides additional strengthening, which offsets part of the negative effect of Laves phase coarsening; the wear rate of 1.33 × 10^−4^ mm^3^·N^−1^·m^−1^ is therefore marginally higher than that of HT500 but substantially lower than HT400, reflecting the benefit of microstructural homogenization even when precipitate coarsening has partially degraded the precipitation-strengthening contribution. The average size of Laves phase precipitates increases from 4.37 μm at HT500 to 4.50 μm at HT600. Such obvious coarsening evidently reduces the number density of effective dislocation-pinning sites and weakens the Orowan precipitation strengthening effect, correspondingly resulting in a moderate hardness reduction of 4.0% and a slight wear rate increase of 9.9% relative to the HT500 specimen.

### 4.6. Synergistic Regulation by Nb Content and Heat Treatment

The effects of Nb content and post-spray annealing on the microstructure and properties of CoCrFeNiNb*_x_* coatings are not independent: the two variables interact through a shared mechanism—the precipitation behavior of the Cr_2_Nb-type Laves phase—such that the benefit of annealing depends critically on the Nb supersaturation established during spraying.

The two variables’ roles are complementary but distinct. Composition control (Nb content) determines the initial precipitation potential; it sets the Nb supersaturation level and the as-sprayed FCC-to-Laves ratio, thereby defining the upper limit of achievable precipitation strengthening and the coating’s baseline toughness. Thermal activation (annealing temperature) then converts this stored potential into actual microstructural improvement, it governs the diffusion kinetics that control precipitate nucleation, growth, and spatial uniformity, while simultaneously relieving residual stresses and closing inter-lamellar porosity. The synergy arises because neither variable alone can achieve the optimal outcome. Nb content without annealing leaves the supersaturation unrealized, while annealing without sufficient Nb supersaturation (e.g., at *x* = 0.25) has little precipitation driving force to act upon.

Among the compositions studied, *x* = 0.75 yields the most favorable as-sprayed starting point: enough Nb supersaturation to drive substantial precipitation strengthening on annealing, yet an FCC-to-Laves ratio that keeps toughness intact. Annealing at 500 °C for 2 h then brings Laves phase precipitation to its optimum and closes residual pores, lifting both hardness and wear resistance to their peak values. This two-step strategy—composition selection followed by controlled low-temperature annealing—provides a practical and substrate-compatible process route for deploying HEA protective coatings on ductile iron components in demanding wear environments. This confirms that the synergistic effect of Nb content and annealing temperature is achieved by precisely regulating the precipitation of Laves phase, and the optimal regulation window is *x* = 0.75 + 500 °C annealing for 2 h.

In summary, increasing Nb content monotonically increased the Laves phase volume fraction and coating hardness, yet wear resistance peaked at *x* = 0.75; beyond this, excess Laves phase degraded fracture toughness and promoted interfacial debonding. Post-spray annealing converted the Nb supersaturation into more uniformly distributed precipitates; within the 400–600 °C window, 500 °C for 2 h proved optimal, as higher temperatures drove coarsening that partially offset the precipitation strengthening effect. On balance, *x* = 0.75 with 500 °C annealing achieved the best trade-off between hardness and toughness.

## 5. Conclusions

(1) CoCrFeNiNb*_x_* (*x* = 0.25–1.00) high-entropy alloy coatings were successfully fabricated on QT800-5 ductile iron via APS. Increasing Nb content induced a microstructural evolution from a single-phase FCC solid solution (*x* = 0.25) to dual-phase microstructure consisting of FCC matrix and Cr_2_Nb-type Laves phase (*x* ≥ 0.50), and the microhardness increased monotonically with Nb addition, reaching 470.36 HV at *x* = 1.00.

(2) The *x* = 0.75 as-sprayed coating exhibited the optimal tribological performance with the lowest wear rate of 2.23 × 10^−4^ mm^3^·N^−1^·m^−1^, which originated from the synergistic balance between Laves-phase strengthening and intrinsic ductile toughening of the FCC matrix.

(3) Among the designed annealing temperatures (400–600 °C), 500 °C annealing for 2 h achieved the best comprehensive performance, delivering a superior hardness of 477.45 HV and a markedly reduced wear rate of 1.21 × 10^−4^ mm^3^·N^−1^·m^−1^.

(4) No obvious microstructural deterioration was observed in the QT800-5 substrate after low-temperature annealing, demonstrating the good process compatibility of the current heat treatment route for ductile iron substrates.

(5) This work clarifies the relationship between Nb alloying, phase evolution, strengthening mechanism, and tribological behavior of plasma-sprayed HEA coatings, and provides feasible technical guidance for surface protection of thermally sensitive ductile iron components. Future work will employ EBSD and TEM for nanoscale precipitate characterization, include residual stress measurements by the sin^2^ψ method, and extend the tribological evaluation to different annealing durations and service-simulated conditions.

## Figures and Tables

**Table 1:** Nominal and measured bulk compositions of CoCrFeNiNb*x* coatings (at.%, O-excluded, area-averaged EDS over 3 fields of 200 × 150 μm per field).

Coating	Nominal Nb (at.%)	Measured Nb (at.%)	Nb Retention (%)
CoCrFeNiNb_0.25_	5.6	4.8 ± 0.6	85.7
CoCrFeNiNb_0.50_	11.1	9.3 ± 1.1	83.8
CoCrFeNiNb_0.75_	15.8	13.1 ± 0.9	82.9
CoCrFeNiNb_1.00_	20.0	15.8 ± 1.4	79.0

**Table 2:** EDS results (at.%) of the marked areas in Figure 4, with O-corrected compositions for points where O > 5 at.%.

Coatings	Positions	Co (at.%)	Cr (at.%)	Fe (at.%)	Ni (at.%)	Nb (at.%)	O (at.%)	Co_corr (at.%) ^1^	Cr_corr (at.%) ^1^	Fe_corr (at.%) ^1^	Ni_corr (at.%) ^1^	Nb_corr (at.%) ^1^
CoCrFeNiNb_0.25_	Point1	7.5	7.7	6.8	6.6	64.3	7.1	8.1	8.3	7.3	7.1	69.2
	Point2	9.9	10.4	15.8	6.3	14.0	43.6	17.6	18.5	28.1	11.2	24.9
	Point3	24.0	22.8	23.7	24.1	4.0	1.5	24.4	23.2	24.1	24.5	4.1
CoCrFeNiNb_0.50_	Point4	7.3	7.1	6.4	7.2	62.7	9.3	8.0	7.8	7.0	7.9	68.9
	Point5	7.3	36.9	9.5	6.9	14.1	25.2	9.8	49.4	12.7	9.2	18.9
	Point6	22.5	23.8	23.1	22.3	8.2	0.2	22.5	23.8	23.1	22.3	8.2
CoCrFeNiNb_0.75_	Point7	5.5	5.3	5.4	5.6	60.7	17.4	6.7	6.4	6.5	6.8	73.5
	Point8	20.5	19.0	20.0	19.8	10.6	10.0	22.8	21.1	22.2	22.0	11.8
	Point9	13.1	31.8	13.7	11.9	14.0	12.5	15.0	36.3	15.6	13.6	16.0
CoCrFeNiNb_1.00_	Point10	20.5	23.4	20.8	20.5	12.0	2.8	21.1	24.1	21.4	21.1	12.4
	Point11	5.1	4.9	5.3	5.0	43.4	36.3	8.0	7.7	8.3	7.8	68.1
	Point12	9.8	24.6	11.8	9.5	20.0	24.4	13.0	32.5	15.6	12.6	26.4

^1^ O-corrected values were calculated by excluding oxygen content and renormalizing the metallic elements to 100 at.% for points with O > 5 at.%.

**Table 3:** Chemical composition (at.%) of the marked points in Figure 9.

Coatings	Positions	Co (at.%)	Cr (at.%)	Fe (at.%)	Ni (at.%)	Nb (at.%)	O (at.%)
CoCrFeNiNb_0.25_	Point1	8.2	8.0	7.9	8.7	1.8	65.5
CoCrFeNiNb_0.50_	Point2	10.9	12.0	11.5	10.6	3.7	51.3
CoCrFeNiNb_0.75_	Point3	5.1	10.2	5.1	4.9	9.7	65.0
CoCrFeNiNb_1.00_	Point4	9.5	11.7	9.5	9.1	14.7	45.5

**Table 4:** Chemical composition (at.%) of labeled points in Figure 11, with O-corrected compositions for points where O > 5 at.%.

Coatings	Positions	Co (at.%)	Cr (at.%)	Fe (at.%)	Ni (at.%)	Nb (at.%)	O (at.%)	Co_corr (at.%) ^1^	Cr_corr (at.%) ^1^	Fe_corr (at.%) ^1^	Ni_corr (at.%) ^1^	Nb_corr (at.%) ^1^
HT400	Point1	5.0	20.0	22.0	5.0	24.7	23.3	6.5	26.1	28.8	6.5	32.2
	Point2	5.0	5.4	5.6	5.1	67.0	10.0	5.6	6.0	6.2	5.7	74.9
	Point3	11.8	18.1	15.8	12.8	10.6	30.8	17.0	26.1	22.8	18.5	15.6
	Point4	22.1	14.4	20.6	22.5	14.1	6.2	23.6	15.3	22.0	24.0	15.1
HT500	Point5	6.5	6.7	6.9	6.5	55.5	18.0	8.1	8.4	8.6	8.1	69.1
	Point6	26.0	10.9	13.4	29.3	20.3	0.0	26.0	10.9	13.4	29.3	20.3
	Point7	20.9	18.8	22.4	21.3	15.2	1.5	21.1	18.9	22.6	21.5	15.3
	Point8	5.9	36.0	18.2	6.5	10.7	22.7	7.6	46.8	23.6	8.4	13.9
HT600	Point9	9.2	20.2	18.3	6.1	23.6	22.6	11.9	26.1	23.7	7.9	30.6
	Point10	12.6	10.7	11.3	13.2	52.1	0.0	12.6	10.7	11.3	13.2	52.1
	Point11	21.4	14.6	16.1	21.1	26.9	0.0	21.4	14.6	16.1	21.1	26.9
	Point12	17.6	22.7	22.3	20.9	16.3	0.1	17.6	22.7	22.3	20.9	16.3

^1^ O-corrected values were calculated by excluding oxygen content and renormalizing the metallic elements to 100 at.% for points with O > 5 at.%.

**Table 5:** Quantitative statistical results of Cr_2_Nb-type Laves phase in CoCrFeNiNb_0.75_ coatings under different annealing conditions.

Annealing Condition	Average Size of Laves Phase (μm, Equivalent Circle Diameter) ^1^	Area Fraction of Laves Phase (%)	Mean Edge-to-Edge Inter-Precipitate Spacing (μm) ^2^
HT0	3.98 ± 0.18	10.79 ± 0.32	5.23 ± 1.12
HT400	3.94 ± 0.15	10.87 ± 0.28	5.18 ± 1.08
HT500	4.37 ± 0.21	16.03 ± 0.45	3.82 ± 0.91
HT600	4.50 ± 0.23	18.65 ± 0.52	3.56 ± 0.88

^1^ The average size is calculated as the equivalent circle diameter from the average area of Laves phase particles measured by ImageJ software, based on the formula *d* = (4*A*/π)^(1/2)^, where *A* is the average area of individual particles. ^2^ The inter-precipitate spacing was calculated using the formula *λ* = *d_avg_* × [(1/*V_f_*)^1/2^ − 1], where *V_f_* is the volume fraction approximated by the measured area fraction. **Note:** All data are presented as the average value ± standard deviation, calculated from 5 non-overlapping BSE-SEM fields of view at 1000× magnification for each sample, with no less than 200 particles counted per field to ensure statistical reliability.

**Table 6:** Chemical composition (at.%) of the marked points in Figure 15.

Coatings	Positions	Co (at.%)	Cr (at.%)	Fe (at.%)	Ni (at.%)	Nb (at.%)	O (at.%)
HT400	Point1	12.5	11.2	13.4	12.2	18.2	32.5
	Point2	7.8	5.6	7.9	7.6	7.0	64.1
HT500	Point3	1.7	2.0	1.8	1.7	50.4	42.5
	Point4	11.2	11.3	11.1	10.6	12.7	44.2
HT600	Point5	12.3	13.8	12.4	11.9	9.3	40.3
	Point6	9.7	10.2	10.7	9.5	6.2	53.6

**Table 7:** Average pearlite inter-lamellar spacing and Vickers hardness of QT800-5 substrate under as-received and annealed conditions.

Specimen State	As-Received	HT400	HT500	HT600
Average lamellar spacing (nm)	164 ± 12	169 ± 8	167 ± 11	174 ± 17
Vickers hardness (HV)	190.35 ± 4.2	190.08 ± 3.8	183.74 ± 4.5	178.92 ± 5.1

## Data Availability

The original contributions presented in this study are included in the article. Further inquiries can be directed to the corresponding author.
